# Women's Ways of Coping With Chronic Widespread Pain—A Phenomenographic Study

**DOI:** 10.1002/msc.70273

**Published:** 2026-10-12

**Authors:** Ingrid Larsson, Katarina Aili, Stefan Bergman, Maria Andersson, Emma Haglund, Ann Bremander

**Affiliations:** ^1^ Spenshult Research and Development Center Halmstad Sweden; ^2^ School of Health and Welfare Halmstad University Halmstad Sweden; ^3^ Department of Clinical Sciences, Section of Rheumatology Lund University Lund Sweden; ^4^ Department of Public Health and Community Medicine Primary Health Care Unit Institute of Medicine The Sahlgrenska Academy University of Gothenburg Gothenburg Sweden; ^5^ Department of Environmental and Biosciences School of Business Innovation and Sustainability Halmstad University Halmstad Sweden

**Keywords:** chronic widespread pain, coping strategies, interviews, phenomenography, qualitative study, women

## Abstract

**Background:**

Chronic widespread pain (CWP) affects around 10% of adults and is more prevalent in women. The impact of CWP on daily life varies, as do coping strategies.

**Objective:**

To describe women's different ways of coping with CWP.

**Methods:**

A descriptive, qualitative phenomenographic study. Semi‐structured interviews were performed with 19 women (aged 45–67) from a population‐based cohort who did not report CWP in 1995 but reported CWP at the 2016 follow‐up assessment. Analysis followed phenomenographic procedures, deriving categories of description and an outcome space.

**Results:**

Four categories described different ways of coping: (i) Taking control by using self‐initiated adjustments, listening to bodily signals, boundary‐setting, and planned recovery; (ii) Continuing as usual by preserving routines and roles while accepting or downplaying pain; (iii) Relying on guidance by using professional advice, structured routines, and tailored programmes; and (iv) Resting by prioritising rest and reducing demands when pain and fatigue dominated. The outcome space showed that these categories were analytically distinct but not mutually exclusive, as women described different ways of coping in different situations depending on bodily signals, resources, and everyday demands.

**Conclusions:**

Women described different ways of coping with CWP in relation to symptoms, everyday demands, and available resources. Recognising this variation may help healthcare professionals provide person‐centred support tailored to current needs, resources, and everyday demands.

## 1 Introduction

Chronic widespread pain (CWP) affects approximately one in ten adults in the general population and is more prevalent among middle‐aged women (Bergman et al. 2001; Fayaz et al. 2016; Mansfield et al. 2016). Living with CWP is linked to substantial burden, including fatigue (Glette et al. 2024), sleep disturbances (Jain et al. 2024) and cognitive difficulties (Bell et al. 2018; Dass et al. 2023), lower physical function (Jaremo et al. 2017; Sylwander et al. 2020), long‐term sickness absence (Mose et al. 2016), participation restrictions (Jaremo et al. 2022; Wang et al. 2020) and worse health‐related quality of life (Jaremo et al. 2017; Sylwander et al. 2020). Clinical guidance for fibromyalgia, in which CWP is a core feature, emphasises education, exercise, and self‐management as first‐line non‐pharmacological approaches, while psychological therapies, such as cognitive behavioural therapy (CBT), selected pharmacological options, and multimodal rehabilitation may be considered according to symptom severity and disability (Macfarlane et al. 2017). Recent fibromyalgia research suggests that reductions in avoidance, catastrophising, and heightened attention to pain signals may underlie symptom improvements during CBT (Hedman‐Lagerlof, Buhrman, et al. 2024a; Hedman‐Lagerlof, Gasslander, et al. 2024b), although systematic reviews and meta‐analyses indicate that treatment effects are often modest (Macfarlane et al. 2017).

In a longitudinal cohort covering 2 decades, approximately three out of four people with persistent CWP were women (Aili et al. 2021). Previous research shows that coping patterns and functioning in chronic pain may differ between women and men (El‐Shormilisy et al. 2015). Women's experiences of coping with CWP may therefore warrant specific attention, particularly as pain experiences, coping and healthcare encounters may be shaped by gendered norms that can influence how pain is expressed, interpreted and treated (Samulowitz et al. 2018). Previous research suggests that experiences of living with chronic pain and the coping strategies used in everyday life vary considerably (Bruce et al. 2023; Hamama and Itzhaki 2023). A widely used definition of coping describes it as the cognitive and behavioural efforts people employ to manage the internal and external demands of situations they appraise as stressful (Folkman and Moskowitz 2004; Lazarus and Folkman 1984). Daily coping strategies in chronic pain may depend on the broader pain experience and the person's journey towards acceptance (Bruce et al. 2023). Contemporary intervention research also highlights acceptance‐based approaches, including effects on pain acceptance, pain‐related functioning, pain interference, and psychological inflexibility (Ma et al. 2023; Ye et al. 2024), alongside activity pacing as a feasible approach to support symptom management (Antcliff et al. 2021).

Although CWP is associated with negative effects across many aspects of life, clinical recommendations and broad coping distinctions provide limited insight into how women themselves understand and manage CWP in everyday life. Such understanding may help healthcare professionals recognise the coping approaches women already use, the situations in which these approaches may support or constrain everyday life, and how support can be adapted accordingly. Given the overrepresentation of women among those with persistent CWP and the potential influence of gendered norms on pain and coping, the aim of this study was to describe women's different ways of coping with CWP.

## 2 Method

### 2.1 Design

This descriptive qualitative study took a phenomenographic approach. Phenomenography adopts a second‐order perspective to describe the qualitatively different ways in which a group experiences or understands a phenomenon. In this study, the phenomenon comprised women's ways of understanding and enacting coping with CWP. Findings are presented at the collective level as categories of description and their outcome space (Marton and Booth 1997). The study adhered to the COREQ criteria for reporting qualitative research (Tong et al. 2007).

### 2.2 Setting

Participants were recruited from among individuals participating in the EPIPAIN study, a prospective population study initiated in 1995 (follow‐up surveys in 1998, 2003, 2007, 2016) to investigate the prevalence and risk factors for long‐term musculoskeletal pain in southern Sweden (Aili et al. 2021; Bergman et al. 2001). The following questions were used to identify CWP: Have you experienced pain lasting more than three months during the last 12 months? If yes (indicating chronic pain), the respondent reported location and distribution of the pain on a manikin with 18 predefined regions; head and abdomen were not included (Bergman et al. 2001, 2002). CWP was identified using the widespread pain distribution component of the 1990 ACR classification criteria for fibromyalgia, pain present in both the left and right sides of the body, above and below the waist, and in the axial skeleton (Wolfe et al. 1990). No tender point examination was performed. Therefore, participants were not classified as fulfiling the full 1990 ACR criteria for fibromyalgia.

### 2.3 Participants

In total, 92 women aged 41 or older at the 2016 follow‐up assessment did not report CWP in 1995 but reported it in 2016. This sampling frame was chosen to include women who had transitioned from not reporting CWP at baseline to reporting CWP at follow‐up, thereby enabling exploration of coping with CWP. From these 92 women, 43 were strategically selected and invited to achieve variation in age, socioeconomic status, educational level, immigration status, smoking habits, alcohol use, social support, heredity for pain, and number of pain sites. In all, 19 women aged 45–67 years (median age 57 years) agreed to participate. Reasons for non‐participation were not requested or recorded. CWP status was identified from the 2016 follow‐up questionnaire, based on questions about chronic pain and pain distribution using a pain manikin. The 2017 interviews focused on women's experiences of coping with CWP; no additional questionnaire‐based reassessment of CWP status or precise symptom duration was available (Table 1).

**TABLE 1:** Characteristics of the 19 participants.

		*N*	Median (min‐max)
Age^a^, years		19	57 (45–67)
Pain sites (0–18)^b^		19	8 (4–16)
Education^b^			
Primary school		2	
Secondary school		12	
University		5	
Married or cohabitant^b^		14	
Sickness absence/disability pension^b^		5	
Sickness absence/disability pension due to pain^b^		3	

^a^ At the time of the interview (2017).

^b^ Information retrieved from the 2016 questionnaire.

## 3 Data Collection

Individual interviews were conducted by authors IL and KA between May and November 2017. Participants were offered flexibility regarding the interview setting and could choose either a digital interview or an in‐person interview at a suitable, undisturbed location. All participating women chose to be interviewed in an undisturbed room at a research centre. At the outset, the study aim, voluntary participation, and the participants' right to withdraw at any time were clarified. A semi‐structured, open‐ended interview guide was developed to ensure comparability across participants (Kvale and Brinkmann 2014). Opening questions included ‘What does CWP mean for you?’; ‘How do you perceive your CWP?’; ‘How do you cope with CWP?’; and ‘Can you describe what you do to influence your CWP?’. Prompts encouraged elaboration (e.g., ‘Please tell me more’, ‘How do you mean?’, ‘What do you have in mind when you say…?’). Two pilot interviews were conducted to test whether the interview guide generated relevant and sufficiently rich descriptions of coping with CWP. The two pilot participants met the same inclusion criteria as the other participants. After the pilot interviews, the content and depth of the interviews were reviewed and discussed within the multidisciplinary research team, which agreed that no changes to the interview guide were needed. The pilot interviews were therefore included in the analysis. The interviews lasted 25–96 minutes (median 58 min). The total interview time was 18 hours and 35 minutes. All interviews were digitally recorded and transcribed verbatim.

## 4 Data Analysis

A phenomenographic analysis was undertaken to qualitatively describe different ways in which women understood coping with CWP. The analysis was guided by phenomenographic principles (Dahlgren and Fallsberg 1991; Sjostrom and Dahlgren 2002). First, the transcripts were read repeatedly while listening to the audio files to obtain an overall understanding, become familiar with the material, and preserve the context of each interview. Second, answers and statements related to coping with CWP were identified and compiled across the entire dataset. Third, these statements were condensed to capture central aspects of how coping with CWP was understood, while retaining their relation to the whole interview. Fourth, similar statements were preliminarily grouped based on similarities and differences in how coping with CWP was understood. Fifth, the preliminary groups were compared and refined to establish analytical boundaries between them. Sixth, the groups were named and formulated as categories of description, ensuring that each category was analytically distinct and defined at the same level of description. Taken together, this iterative process yielded four categories of description of coping with CWP: To take control, To continue as usual, To rely on guidance, and To rest. Seventh, the categories were contrastively compared, and the relationships among them were articulated in the outcome space (Figure 1). For example, statements in which a woman described listening to bodily signals, setting boundaries, planning recovery, and combining personal responsibility with professional input were initially interpreted as self‐initiated adjustment. Through comparison with other accounts, this interpretation contributed to the category of description ‘To take control’ and was positioned in the outcome space as a way of coping characterised by self‐directed initiative, bodily awareness, and planned adjustment. The primary analysis was conducted by the first, second, and last authors (IL, KA, and AB). Analytical decisions were finalised through a negotiated consensus process within the multidisciplinary research team, which comprised representatives from nursing (IL), physiotherapy (KA, EH, and AB), medicine (SB), and biomedical laboratory science (MA).

### 4.1 Ethics Statement

This research conforms to the Declaration of Helsinki (WMA 2024) and fulfils the requirements for information, consent, confidentiality, and participant safety (Swedish Research Council 2024). Ethical approval was obtained from the Ethical Review Board in Lund, Sweden (Reg. No. LU 389‐94, 2016/132, 2016/786). Participation was voluntary, informed consent was obtained, and participants could withdraw at any time. Personal data were registered according to the General Data Protection Regulation (GDPR2016/679) and stored in accordance with Sweden's Archive Act (SFS1990:782).

## 5 Results

The result comprised four categories of description, representing different ways in which women understood coping with CWP: To take control, focusing on self‐initiated adjustments in everyday life; To continue as usual, focusing on preserving normality despite pain; To rely on guidance, focusing on professional guidance and structured support; and To rest, focusing on recovery when pain and fatigue dominated. Each category was illustrated with quotations. The relationships among the categories are summarised in the outcome space.

### 5.1 To Take Control

To take control reflected an understanding of coping with CWP as managing everyday life in ways that helped maintain balance. Coping was described as making deliberate decisions and self‐initiated adjustments, guided by knowledge, confidence, and awareness of the body's signals. By listening to bodily needs and planning recovery, women described creating a workable rhythm between activity and rest. This involved setting boundaries to protect well‐being and adjusting strategies in response to bodily signals and everyday demands. Responsibility for managing CWP was understood as shared with healthcare professionals, combining personal agency with professional input.

To acquire knowledge, quite simply […] and also to receive practical advice from the physiotherapist […] and to piece all these parts together into what suits me. I do those movements, I get that bicycle, and I take responsibility […] after all, it is my life […] it is I who have to change what can be changed […] I start with one thing, then when that works, you add something else […] it really is a process.

(Participant no. 3)

Taking control was also described as prioritising physical and psychosocial needs: staying active at a sustainable level, protecting the body, cultivating conditions for sleep and recovery, and focusing on sources of energy while distancing from ‘energy thieves.’ A positive mindset, supportive relationships, and time in nature were described as resources that were intentionally used to support coping with CWP in everyday life.

You simply do things that you enjoy […] You come to realise that you can’t just sit on a chair and think the pain will disappear. You need to keep moving; the more active I am, the better I feel […] and you surround yourself with people who give something back […] you take care of yourself […] live your own life, because this is where and when you are living.

(Participant no. 2)

In this category of description, to take control was understood as coping with CWP by listening to bodily signals, making adjustments, and establishing daily routines. This approach was characterised by boundary‐setting, planned recovery, and iterative refinement of strategies, and was understood to enable meaningful engagement in work and leisure. To take control and adapting to the challenges of living with CWP created a sense of stability. The ability to adjust and to establish routines was understood as reflecting a deep understanding of how to cope with CWP.

### 5.2 To Continue as Usual

Continue as usual reflected an understanding of coping with CWP as preserving normality and everyday continuity by keeping pain in the background. Coping was described as being centred on keeping work, routines, and roles going, and looking forward rather than dwelling on difficulties. Maintaining daily routines, such as working, walking, running, and other leisure activities, was described as the key to preserving this continuity. Some chose not to talk about chronic pain to avoid having their experiences dismissed or misunderstood. Acceptance of life with CWP was described as enabling momentum and maintaining everyday function.

I don’t want to be in pain […] I’ve had a strategy of not thinking about it. I decided that it shouldn’t affect my life. I want to move forward […] to accept that this is how it is. I do what I always do […] maybe take an extra tablet, and then I move on […] I carry on as usual.

(Participant no. 9)

Women described being consistently present for others at work and at home, and being trusted to step in when needed. Reluctance to ask for help and a tendency to take on tasks were described as increasing responsibilities and blurring boundaries. Continuing life as usual often involved downplaying or ignoring bodily signals, to keep routines intact. In pushing through pain to preserve normality, other symptoms were at risk of being overlooked. This created a tension between maintaining normality and continuing beyond one's limits. This way of coping was described as prioritising function and continuity in everyday life while coping with CWP, sometimes with the aim of maintaining life as it was before the onset of CWP.

Then it (the pain) is more of a companion, a stowaway you would like to drop off […] You grit your teeth, forget about it […] you accept your situation […] everything becomes easier once you have accepted it […] you get used to it. I’m not the type to dwell and wonder why I’m in pain […] it’s just the way it is […] otherwise, everything just becomes more miserable overall.

(Participant no. 8)

In this category of description, To continue as usual was understood as coping with CWP by upholding everyday life despite pain. This approach was characterised by maintaining routines and social roles, accepting or downplaying pain, and keeping bodily signals in the background in order to preserve continuity in work, relationships, and daily responsibilities. The commitment to continue as usual was understood as reflecting a way of coping in which normality and moving forward were prioritised, even when this could involve continuing beyond one's limits.

### 5.3 To Rely on Guidance

Relying on guidance reflected an understanding of coping with CWP as gaining stability through professional advice, structured programmes, and trust in healthcare professionals. Coping centred on using professional recommendations and incorporating tailored programmes into everyday life. Responsibility for planning and structuring treatment was described as resting largely with healthcare professionals, while the women described carrying out the routines within their own everyday circumstances. Confidence in independent decision‐making was described as limited, and tailored, structured guidance was preferred.

I can feel that I want someone who has tailored something for me […] then you can be free. I have a physiotherapist […] once a week […] and they set up a programme.

(Participant no. 12)

Maintaining routines and seeking personalised exercise programmes were described as supporting coping with CWP in everyday life. Some engaged trusted personal trainers and used recommendations, integrating professional advice into everyday life to support activity and recovery within the context of CWP.

The physiotherapist has listened and designed exercises that suit me […] You can’t just sit around feeling sorry for yourself […] I have to do a little myself, whatever I am able to manage. We set it up so […] start running a little bit […] He’s the one who got me started.

(Participant no. 14)

In this category of description, To rely on guidance was understood as coping with CWP through professional guidance, structured routines and tailored support. Coping was characterised by seeking tailored programmes and incorporating professional input to plan activity and recovery. The resulting predictability was understood to foster stability.

### 5.4 To Rest

Rest reflected an understanding of coping with CWP as relying on rest and recovery when pain and fatigue dominated everyday life. Rest was described both as intentional self‐protection and as a necessary response when symptoms reduced the possibility of maintaining usual activities. Pain and fatigue dictated daily life; planned activities were cancelled and time at home was prioritised. Leisure was reduced, and recovery consumed available energy, leading to resignation.

What I really need is probably more rest… quite often, someone like me ends up skipping certain things because they feel, no, I don’t have the energy. I’m tired, I can’t manage. I’d rather stay at home and take it easy quite a lot instead of doing things… Sometimes I just want to go to bed; on a few occasions, I have actually left guests and gone to lie down.

(Participant no. 16)

Coping with CWP was described as a negative spiral in which persistent pain led to fatigue and fatigue exacerbated pain. This spiral was understood as draining energy and fostering a sense of powerlessness. Rest and relaxation were described as essential strategies for protecting energy and preventing further exhaustion. At the same time, the need for recovery restricted daily activities and diminished the quality of life, reinforcing the experience of being controlled by pain.

You get really tired, weak, and low on energy […] it’s important to rest, otherwise you won’t manage at all […] if you don’t get rest and sleep […] you also get more pain, it becomes like a spiral […] You tend to think a bit more about yourself and make sure you feel well, taking time to rest, take it easy, and not just push on.

(Participant no. 18)

In this category of description, To rest was understood as coping with CWP by prioritising rest when pain and fatigue dominated everyday possibilities. Rest was described as a way of protecting energy, and managing symptoms, but also as restricting activities when recovery consumed available energy. This approach was characterised by reducing demands and stepping back from activities when symptoms left little room for maintaining everyday routines.

## 6 Outcome Space

The outcome space described how the four categories of description related to one another in this study (Figure 1). The categories were organised according to variation in how women understood coping with CWP, the source of structure, and the orientation of coping in everyday life. Taking control was characterised by self‐directed adjustment, bodily awareness, and planned recovery. Continuing as usual was characterised by preserving normality and everyday continuity, often by keeping pain in the background. Relying on guidance was characterised by professional guidance and structured support as sources of stability. Resting was characterised by conserving energy and reducing demands when pain and fatigue dominated.

**FIGURE 1:**
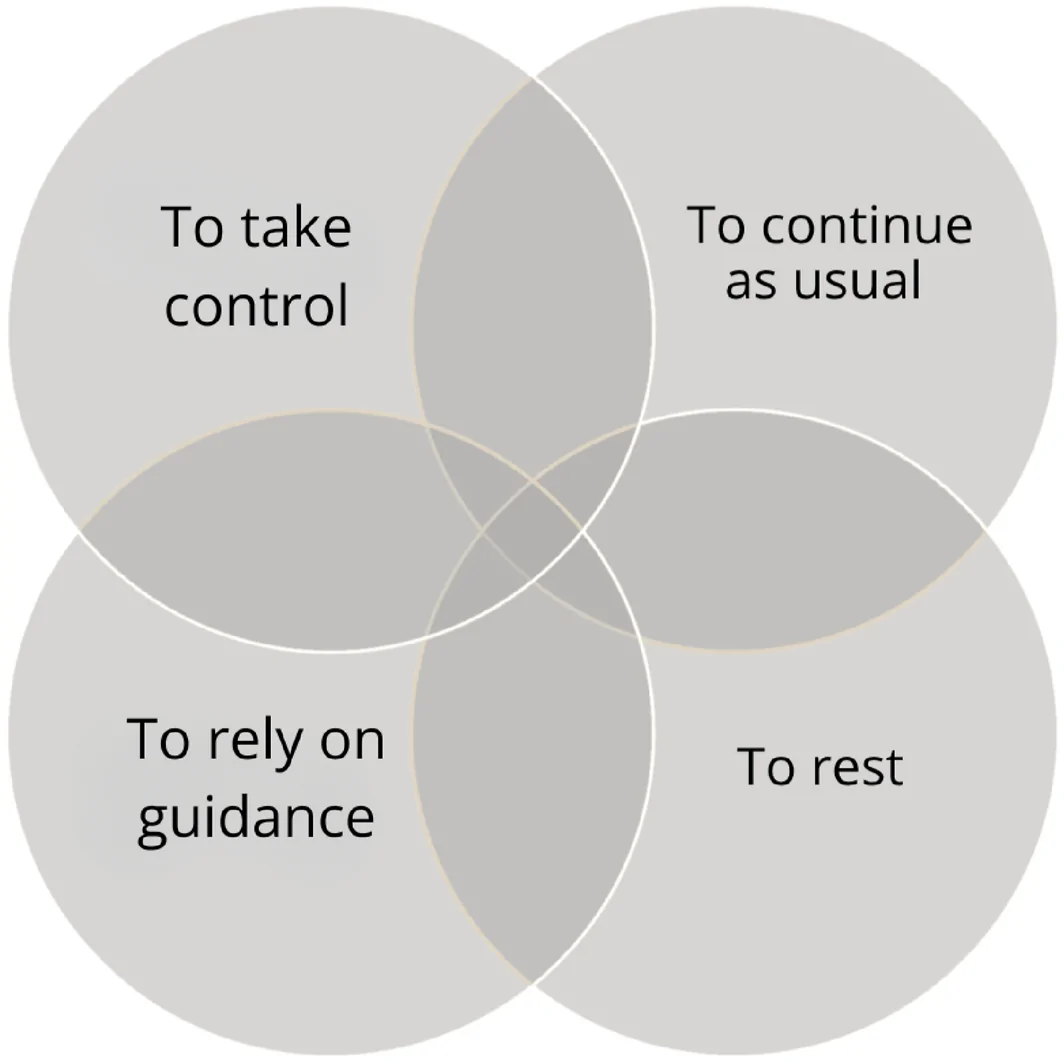
Outcome space showing four categories of description of coping with chronic widespread pain (CWP), organised according to variation in the source of structure and the orientation of coping in everyday life. Overlaps indicate that the categories are analytically distinct but not mutually exclusive, and that women could describe more than one way of coping depending on symptoms, resources, and everyday demands.

The four categories of description were defined at the same level of description and were analytically distinct. However, the categories should not be understood as fixed positions, as mutually exclusive ways of coping in everyday life, or as indicating movement or change in coping over time. Rather, they represent different ways in which women described coping with CWP in relation to bodily signals, available resources, and everyday demands. The outcome space, therefore, displays value‐neutral variation in ways of coping with CWP, rather than a hierarchy between categories.

## 7 Discussion

In this study, four different ways of experiencing and enacting coping with CWP were identified: taking control, continuing as usual, relying on guidance, and resting when pain and fatigue dominate. These ways of coping were described in relation to symptoms, everyday demands, and available resources. The findings show that coping with CWP may involve self‐directed action, maintaining everyday continuity, relying on professional guidance, and conserving energy through rest. People with chronic pain report drawing on a suite of coping options that varies by day and circumstances along a journey to acceptance (Bruce et al. 2023).

Taking control and continuing as usual both involved efforts to sustain participation in everyday life, but differed in how bodily signals and everyday demands were handled. Taking control was characterised by self‐directed adjustment, boundary‐setting, attention to bodily signals, and planned recovery. These aspects align with previous research describing activity pacing as the regulation of activity level or rate to support adaptive goals, symptom management and engagement in meaningful activities (Antcliff et al. 2021; Nielson et al. 2013). Continuing as usual, by contrast, was oriented towards preserving normality and everyday continuity, sometimes by downplaying pain signals or continuing beyond one's limits. This way of coping may support participation, identity, and valued roles, but should not be interpreted as inherently adaptive or as equivalent to pacing. Rather, its implications may depend on the situation, symptom load, available resources, and whether maintaining continuity also allows sufficient recovery. This more nuanced interpretation is consistent with research on task‐contingent persistence and overactivity in chronic musculoskeletal pain, where persisting with activity may have different consequences depending on context, recovery, and symptom regulation (Burrus et al. 2020; Luthi et al. 2018). Conceptually, flexible use of different ways of coping can be understood in relation to psychological flexibility, where functioning and participation are supported through values‐guided action, acceptance, and adaptation to current circumstances (Gentili et al. 2019; Ma et al. 2023; McCracken 2024; Ye et al. 2024). Acceptance was one aspect of continuing as usual, but may also be understood as supporting the use of different ways of coping depending on the situation, in line with research describing acceptance as part of coping with chronic pain (Bruce et al. 2023) and as associated with better pain‐related function over time (Jensen et al. 2016).

Relying on guidance and resting reflected different ways in which coping was structured when symptoms, uncertainty, or limited resources shaped everyday life. Relying on guidance reflected trust in professional guidance and structured programmes, while planning and structuring of treatment were described as largely professionally led. This should not be understood as behavioural passivity or lack of agency, but as a way of coping in which structure and decision‐making were supported by professional input. Resting involved prioritising rest and reducing demands when pain and fatigue dominated everyday life. Rest could be understood as intentional recovery and energy conservation, but also as restricting activities when recovery consumed available energy. As coping strategies, these approaches may provide short‐term stabilisation when symptom burden is high, particularly when professional guidance, planned rest, and energy conservation help to preserve function and prevent further deterioration (Kim et al. 2022). This is consistent with fatigue self‐management research, where strategies, active patient participation and self‐management support are described as central components of programmes for people living with chronic conditions (Alizadeh et al. 2023). For externally guided or restorative coping to support everyday participation, it may be important that such support is linked to agreed goals, planned activities, and opportunities for re‐engagement rather than remaining solely symptom‐contingent or dependent on external structure. External guidance and rest can then function as pragmatic starting points for planned and graded re‐engagement. This may involve adjustment, planning and progression of activities (Antcliff et al. 2021) using pre‐set time or amount targets and stepwise increases rather than symptom‐contingent stopping (Nielson et al. 2013). Relying on guidance may therefore be understood as potentially supportive when professional input is translated into regular, predictable routines, agreed activity levels and planned progression. Exercise studies in people with widespread pain conditions indicate that appropriately dosed and progressively increased exercise may contribute to improvements in pain, disease impact and health‐related quality of life (Albuquerque et al. 2022; Han et al. 2024; Rodriguez‐Almagro et al. 2023). In this way, professionally guided routines may provide structure for more regular participation in everyday life, particularly when combined with personalisation, group support, and planned post‐intervention support that helps self‐management strategies be applied in daily life (Geraghty et al. 2021; Hu et al. 2025). Taken together, relying on guidance and resting may support coping when they are linked to recovery, structure, and possibilities for re‐engagement. In this way, guidance‐based coping can support graded re‐engagement, while rest can be understood as recovery that enables renewed participation rather than as an endpoint in itself (Antcliff et al. 2021). When professional guidance or rest is linked to meaningful participation, these ways of coping may be understood in relation to processes of psychological flexibility (Ma et al. 2023; Ye et al. 2024). However, if externally guided or restorative coping becomes long‐term and symptom‐contingent, it may risk reinforcing avoidance or rigid endurance, with consequences for everyday participation (Rogers and Farris 2022; Vlaeyen et al. 2016).

### 7.1 Practical Implications

Taken together, for women with CWP, these findings provide a basis for person‐centred support: start from how each woman currently copes in different situations, co‐define valued goals and constraints, and use time‐contingent, graded steps with follow‐up support to sustain participation (Antcliff et al. 2021; Geraghty et al. 2021; Hu et al. 2025; Ma et al. 2023; Ye et al. 2024). Care should be a shared responsibility between women and healthcare professionals. Discussing how coping differs across symptoms, demands, and resources may support psychological flexibility, self‐management, and person‐centred support that is tailored to each woman's situation. In practice, interventions should meet each woman where she is. This includes providing graded, confidence‐building support for self‐directed coping when appropriate, while also recognising that some women may need or prefer more structured professional guidance. In line with a person‐centred approach, co‐created health plans for physical activity in people with CWP can incorporate personal goals, resources, and support needs, and facilitate engagement in physical activity (Larsson et al. 2023). When physical activity is part of a self‐directed way of coping, recommendations should be tailored to each woman's resources and situation. Evidence from exercise studies supports attention to appropriate dosing and gradual progression (Albuquerque et al. 2022; Han et al. 2024; Rodriguez‐Almagro et al. 2023). Rest, energy conservation, and professional guidance should also be available when appropriate, with support for re‐engagement when circumstances allow.

### 7.2 Strengths and Limitations

The trustworthiness of the study was evaluated using the criteria of credibility, dependability, confirmability, and transferability (Lincoln and Guba 1985), with particular attention to credibility in phenomenographic analysis (Sjostrom and Dahlgren 2002). In phenomenography, credibility concerns the relationship between the empirical material and the categories of description, including whether the categories are well supported by the participants' accounts. Credibility was supported through purposive sampling, in‐depth semi‐structured interviews, repeated reading of the transcripts, and the use of quotations to show how the categories were grounded in the participants' accounts. A multidisciplinary team conducted reflexive, collaborative analysis and reached a negotiated consensus, bringing diverse perspectives and strengthening interpretations. Participant validation during the interviews was used by summarising and confirming participants' statements to support accurate understanding. Dependability concerns the stability and consistency of the research process over time. Through a consistent interview structure, use of the same semi‐structured interview guide across all interviews, and a documented, stepwise phenomenographic analysis, the dependability was strengthened. Analytical decisions were discussed until the team reached consensus to enhance coherence. The fact that two interviewers conducted the interviews may be seen as both a strength, bringing broader perspectives, and a limitation, due to potential variation in interviewing style. Confirmability refers to the extent to which the findings are grounded in participants' accounts rather than researchers' preconceptions. Confirmability was strengthened through transparent reporting of the methodological approach and analytical steps, verbatim transcription, and the inclusion of participant quotations to anchor interpretations in the data. Transferability concerns the applicability of the findings to other contexts. Transferability is supported by thick descriptions of the study context and participant characteristics, along with sampling for variation within a larger population‐based study in southern Sweden. However, the focus on women within a specific age range and regional context may limit their applicability to other settings. The underlying pain mechanisms were not assessed, and the heterogeneity of CWP should therefore be considered when applying the findings to other chronic pain populations or clinical contexts. The interval between the 2017 interviews and publication should also be considered, as developments in chronic pain science and self‐management support may influence transferability to current practice. Because all participating women chose research‐centre interviews, women with more severe symptoms or transportation difficulties may be underrepresented.

## 8 Conclusions

Women living with CWP described four ways of coping in different situations depending on symptoms, everyday demands, and available resources: taking control, continuing as usual, relying on guidance, and resting. These ways of coping differed in how women related to bodily signals, everyday routines, professional guidance, responsibility, and the need for recovery. Taking control involved self‐initiated adjustments, attention to bodily signals, and sustainable routines, whereas continuing as usual involved maintaining normality in work and everyday roles while accepting or downplaying pain. Relying on guidance involved using trusted professional guidance and tailored programmes, while resting involved prioritising rest and reducing demands when pain and fatigue dominated.

In clinical practice, healthcare professionals should tailor support to how women cope in different situations and provide person‐centred support based on current needs, resources, routines, and limits. This includes strengthening self‐directed strategies such as pacing, boundary‐setting, and routine planning when appropriate, while also providing clear, tailored guidance and structured programmes when external input is preferred. It also involves recognising and legitimising periods of rest and energy conservation, including practical adjustments at work and at home. Further research should address person‐centred support for women with CWP, including family support and tailored guidance, with attention to how coping can be supported in everyday life and how participation can be sustained.

## Author Contributions

All authors were involved in conception and design, as well as in the interpretation and critical revision of the intellectual content. The primary data analysis and manuscript drafting were conducted by IL in collaboration with K.A. and A.B. The final version of the manuscript was approved for publication by all authors, and the authors acknowledged full responsibility for all aspects of the work.

## Funding

Open access funding was provided by Halmstad University. This research did not receive specific funding from any public, commercial, or not‐for‐profit agency.

## Conflicts of Interest

The authors declare no conflicts of interest.

## Data Availability

The datasets generated during and/or analysed during the current study are not publicly available due to legal and ethical restrictions surrounding participant confidentiality. De‐identified excerpts of the data can be made available from the corresponding author upon reasonable request. Requests for data access may be subject to approval by the relevant ethics committee. For further information, please contact the corresponding author.
